# The prevalence of uterine fundal pressure during the second stage of labour for women giving birth in health facilities: a systematic review and meta-analysis

**DOI:** 10.1186/s12978-021-01148-1

**Published:** 2021-05-18

**Authors:** Elise Farrington, Mairead Connolly, Laura Phung, Alyce N. Wilson, Liz Comrie-Thomson, Meghan A. Bohren, Caroline S. E. Homer, Joshua P. Vogel

**Affiliations:** 1 Maternal, Child and Adolescent Health Program, Burnet Institute, 85 Commercial Road, Melbourne, VIC 3004 Australia; 2 Melbourne Medical School, Faculty of Medicine, Dentistry and Health Sciences, The University of Melbourne, Parkville, VIC 3010 Australia; 3 Gender and Women’s Health Unit, School of Population and Global Health, Centre for Health Equity, The University of Melbourne, Parkville, VIC 3010 Australia; 4 School of Population and Global Health, The University of Melbourne, Parkville, VIC 2010 Australia

**Keywords:** Fundal pressure, Intrapartum care, Kristeller maneuver, Labour and childbirth, Mistreatment during childbirth, Quality of care

## Abstract

**Background:**

Uterine fundal pressure involves a birth attendant pushing on the woman’s uterine fundus to assist vaginal birth. It is used in some clinical settings, though guidelines recommend against it. This systematic review aimed to determine the prevalence of uterine fundal pressure during the second stage of labour for women giving birth vaginally at health facilities.

**Methods:**

The population of interest were women who experienced labour in a health facility and in whom vaginal birth was anticipated. The primary outcome was the use of fundal pressure during second stage of labour. MEDLINE, EMBASE, CINAHL and Global Index Medicus databases were searched for eligible studies published from 1 January 2000 onwards. Meta-analysis was conducted to determine a pooled prevalence, with subgroup analyses to explore heterogeneity.

**Results:**

Eighty data sets from 76 studies (n = 898,544 women) were included, reporting data from 22 countries. The prevalence of fundal pressure ranged from 0.6% to 69.2% between studies, with a pooled prevalence of 23.2% (95% CI 19.4–27.0, I^2^ = 99.97%). There were significant differences in prevalence between country income level (p < 0.001, prevalence highest in lower-middle income countries) and method of measuring use of fundal pressure (p = 0.001, prevalence highest in studies that measured fundal pressure based on women’s self-report).

**Conclusions:**

The use of uterine fundal pressure on women during vaginal birth in health facilities is widespread. Efforts to prevent this potentially unnecessary and harmful practice are needed.

**Supplementary Information:**

The online version contains supplementary material available at 10.1186/s12978-021-01148-1.

## Plain Language Summary

Uterine fundal pressure involves a health worker pushing on the uppermost part of a woman’s abdomen during the pushing phase of labour, with the aim of assisting or accelerating vaginal birth. The World Health Organization and other bodies specifically recommend against the use of fundal pressure, as it is not beneficial and is potentially harmful to women. This study undertook a review to determine how often fundal pressure is used on women giving birth in hospitals around the world. We searched five databases and found 76 studies from 22 countries. We determined that 23.2% of women experience some form of fundal pressure during the pushing phase of labour. Results between studies varied widely, ranging from 0.6% to 69.2% of women experiencing some form of fundal pressure. This may be due to different study populations, or different methods of assessing or documenting fundal pressure use. It may also reflect differences in clinical practice or guidelines. Despite these differences, our findings indicate uterine fundal pressure is still widespread and efforts to prevent this potentially unnecessary and harmful practice are needed.

**Supplementary Information:**

The online version contains supplementary material available at 10.1186/s12978-021-01148-1.

## Background

Maternal mortality and stillbirth continue to be significant issues globally, with an estimated burden of 295,000 maternal deaths and 2.6 million stillbirths occurring worldwide each year [1, 2]. It is estimated that 27.7% of maternal deaths occur during or immediately following birth and 50% of stillbirths occur intrapartum [2, 3]. Quality intrapartum care is essential to optimise maternal, fetal and neonatal peripartum outcomes and experiences of care [4]. Ideally, maternity care practices should reflect the latest evidence and clinical guidelines, however there are recognised gaps between recommended care and actual practice in many settings [5, 6].

Uterine fundal pressure is pressure applied to a woman’s uterine fundus in the direction of the vagina during the second stage of labour with intention to promote or accelerate the time to a spontaneous vaginal birth [7]. With a prolonged second stage of labour, maternal exhaustion may reduce a woman’s ability to generate sufficient abdominal pressure to facilitate her baby’s birth [8–10]. Application of external force through fundal pressure has previously been thought to assist vaginal birth, reducing the need for alternative and more invasive interventions to manage prolonged second stage—such as vacuum extraction, forceps delivery or Cesarean section [9]. Additionally, use of fundal pressure in some resource poor settings may be attributed to a lack of access to alternative interventions [5]^43^. While fundal pressure is used during caesarean section to assist expulsive effort (to deliver the fetus when the uterus is not contracted), its use in vaginal birth is more controversial [7]. Methods of fundal pressure application vary, generally involving external manual pressure from a birth attendant. This ranges from gentle pressure to the full force of an attendant's body weight [7, 11]. Excessive force can subject the woman’s uterine fundus to uneven, high-intensity pressure [12]. Targeted devices, such as inflatable abdominal pressure belts, have been used in research settings to apply fundal pressure in a more controlled manner [7].

A 2017 Cochrane review identified five randomised trials using manual uterine fundal pressure [7]. The review found no difference in mode of birth outcomes or duration of second stage of labour for women with manual fundal pressure. More women who received manual fundal pressure had cervical tears, though this was based on findings from a single trial (295 women). The review authors concluded that there was insufficient evidence on the benefits and harms of this procedure. More recently, a trial of 1158 nulliparous women in South Africa used gentle assisted manual pushing during second stage of labour, finding no evidence of benefit but more women reporting discomfort [12].

Some authors have reported concerns on potential harmful outcomes for the woman and baby with the misuse of fundal pressure, such as when excessive force is used [13–18]. Increased risk of adverse events such as perineal damage, shoulder dystocia and neonatal birth injuries in women who receive fundal pressure has been reported in observational studies [13–18]. Additionally, use of fundal pressure may result in reduced women’s satisfaction with the labour and birth experience, and could decrease the likelihood of the woman engaging with skilled health personnel in future births [11, 19].

The World Health Organization (WHO) and several other national obstetric societies specifically recommend against the use of fundal pressure [20–23]. Despite these recommendations, there are reports of routine fundal pressure use during vaginal birth [11, 12], however the prevalence of its use internationally has not been determined. The aim of this study was to determine the prevalence of uterine fundal pressure during the second stage of labour for women giving birth vaginally in health facilities.

## Methods

This systematic review was conducted in accordance with the Preferred Reporting Items for Systematic Reviews and Meta-Analyses (PRISMA) guidelines (Additional file 1) [24]. The study protocol was registered with PROSPERO (CRD42020169126). Ethics approval was not sought as this was a systematic review of publicly available data.

### Eligibility criteria

Any primary study using an observational or interventional design was eligible. This included case–control, cohort, cross-sectional and descriptive studies, as well as quasi-randomised or randomised trials. Conference abstracts were included if they provided sufficient information for data extraction. Case reports, case series, letters and commentaries were not included. To focus this review on contemporary maternity care practice, we opted to include only eligible studies published on or after 1 January 2000. Studies published in any language were eligible.

Our population of interest was women who experienced labour in a health facility, in whom vaginal birth was anticipated. Some of these women may have then undergone intrapartum caesarean section. Women of any age, ethnicity, parity or gestation from any country were included. Studies pertaining only to women giving birth outside of health facilities (such as at home or in community settings) were not included. We excluded studies in the use of fundal pressure during caesarean section, during third stage or as part of shoulder dystocia management. Women undergoing an elective or non-urgent caesarean section or a caesarean section commenced in the first stage of labour were also excluded.

### Outcomes

The primary outcome for this review was the prevalence of uterine fundal pressure during the second stage of labour. To further explore available data, we stratified results by decade of publication (1991–2000, 2001–2010, 2011–2020), method of fundal pressure application, method of measuring use of fundal pressure (women’s self-report, direct observation of labour, medical records), parity (nulliparous, multiparous) and country income level (low, lower-middle, upper-middle, high) based on the 2020 World Bank Classification [25].

### Search strategy

Studies were retrieved from MEDLINE, EMBASE, CINAHL and Global Index Medicus databases on 14 February 2020 using a pre-specified search strategy that was developed in consultation with an information specialist (Additional file 2). Free text and index terms were adapted to suit each electronic database, comprising the two main search concepts: (a) fundal pressure and (b) second stage of labour. Forward citation searching of previous systematic reviews on the topic (current and previous versions of the Cochrane review on fundal pressure during the second stage of labour) was completed via Google Scholar to obtain further studies [7, 26]. Reference lists of included studies were reviewed to identify any additional, relevant studies.

### Study selection, data extraction and quality assessment

After removing duplicates, two reviewers (EF, LP, MC) independently assessed titles and abstracts of unique citations for inclusion. Full texts were collected for potentially eligible studies, and also reviewed in full by two independent reviewers (EF, LP, MC). Disagreements between reviewers were resolved through discussion or through consultation with a third reviewer. For studies requiring further information to assess eligibility, an attempt was made to contact the authors. Where we were unable to obtain further information to assess eligibility, the study was not included. In the event of identifying two (or more) papers reporting data from the same study population, the paper providing the largest sample size was selected, with duplicate papers excluded. Citations were collated using EndNote X9 [27] and screening was conducted using Covidence [28]. Two independent reviewers (EF, LP, MC) extracted data from eligible studies using a standardised data extraction form that had been pilot-tested on three eligible studies. We extracted details on study characteristics, the prevalence of fundal pressure use, duration of data collection, sampling technique, method of measuring use of fundal pressure, mode of birth, method of fundal pressure application and provider of fundal pressure (if available). Data from each reviewer were reconciled, with any discrepancies resolved through discussion. Data were extracted verbatim, then categorised for analysis.

In order to assess risk of bias each study was assessed using an 8-point checklist (Additional file 3), which was developed by adapting Rotenstein et al.’s Modified Newcastle–Ottawa Scale [29] and Hoy et al.’s tool for population-based prevalence studies [30]. Studies were graded out of eight points, and categorised as low (score 0–2), moderate (score 3–5) or high (score 6–8) quality. Two reviewers (EF, LP, MC) scored each study independently, with results compared and any differences resolved through discussion or through consultation with a third reviewer.

### Data analysis

To determine the prevalence of fundal pressure during the second stage of labour, a meta-analysis was conducted using Stata SE 16.1 [31]. Heterogeneity was assessed using the I^2^ statistic, with a random effects model used where I^2^ was greater than 50%.

Sensitivity and subgroup analyses were conducted to explore potential sources of heterogeneity. Three separate sensitivity analyses were conducted by excluding studies with: (1) a sample size less than 500; (2) studies categorised as low or moderate quality; and (3) studies where the study population included only a subset of women (e.g. women with prolonged second stage of labour). Subgroup analyses were conducted by stratifying studies by decade of publication, method of fundal pressure application, method of measuring use of fundal pressure, parity and country income level.

## Results

**Fig. 1:**
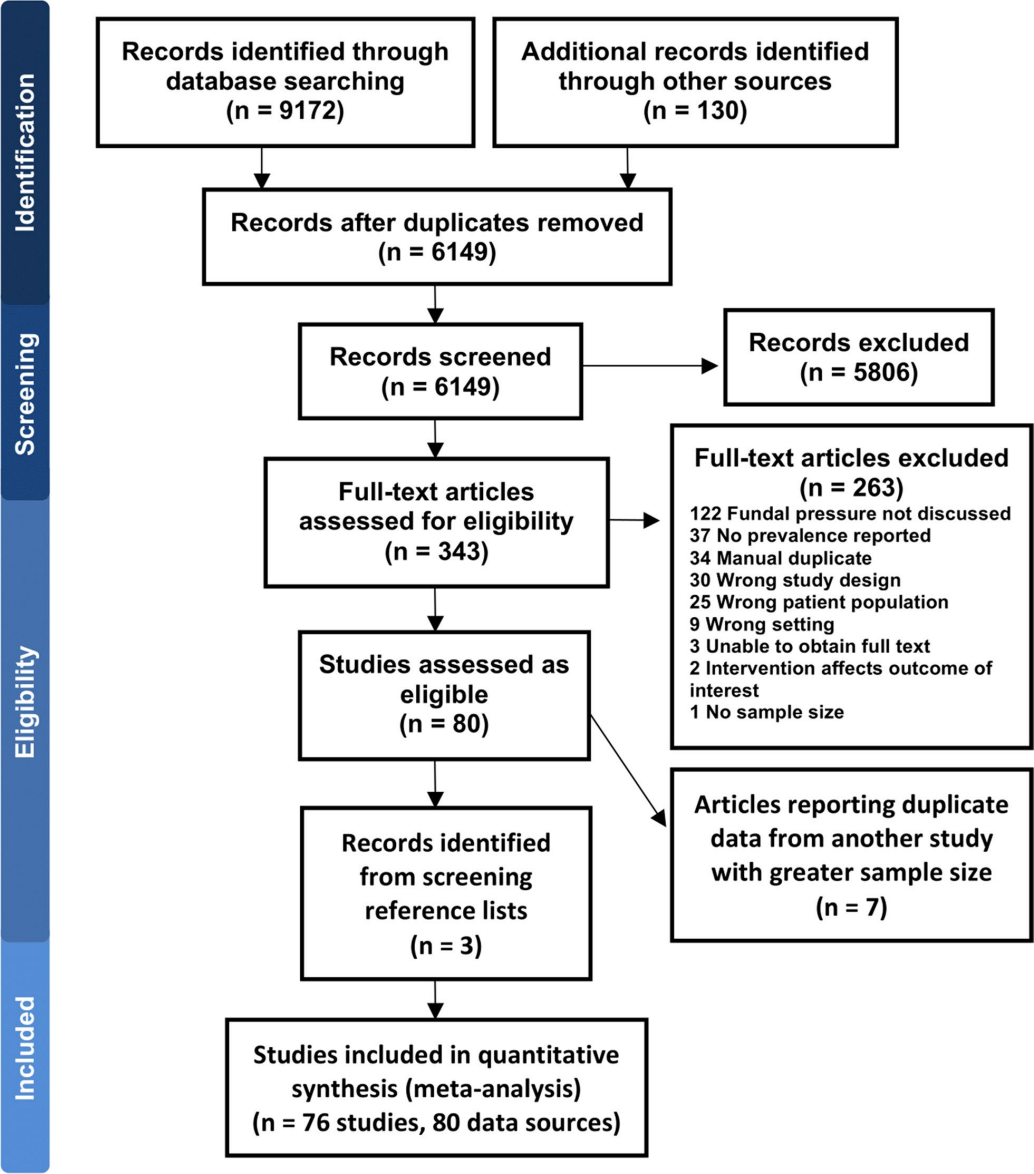
PRISMA flow chart demonstrating inclusion and exclusion of studies

A total of 9172 citations were identified, with a further 130 studies identified through forward citation searching of the Cochrane review [7]. After removal of duplicates, 6149 unique citations were screened and 343 citations identified for full text review (Fig. 1). Full texts were available for 313 studies, with 80 studies included. Reference list screening yielded three additional studies. Seven studies were subsequently identified as reporting the same data as reported in other studies and were excluded. Four of the 76 included studies provided data for two separate datasets, creating a total of 80 data sources used for meta-analysis.

Characteristics of the included studies are reported (Table 1 and Additional file 4). The 80 data sources comprising 898,544 women were conducted across 22 countries (Table 2). 29/76 studies (38%) were conducted in Brazil and Italy, and 762,408/898,544 (84.8%) women were in Japan. Data collection dates ranged from 1994 to 2019. The majority (75/80) of studies had observational designs: with six case–control, 51 cross-sectional, eight descriptive, six prospective cohort and four retrospective cohort studies (Table 3). Five studies had interventional designs: two quasi-experimental before-and-after studies and three randomised controlled trials. Sample sizes ranged from 16 to 404,444 women. The method of measuring use of fundal pressure was based on women’s self-report in 19 studies, direct observation of women in labour (typically by a research assistant) in 29 studies, and derived from medical record review in 24 studies (source not specified in eight studies). Two studies recorded data on fundal pressure use from both woman’s self-report and direct observation in the same study population; in these studies, we opted to preferentially extract data on direct observation only. Data were recorded during labour and childbirth care in most studies (54/80) (Table 3).

**Table 1:** Characteristics of the 80 datasets from 76 included studies

Author Year	Study design	Country	Income level (2020 World Bank)	Method of measuring use of fundal pressure	Last year of data collection	Study population (denominator)	Fundal pressure (numerator)	%	Risk of bias
Abasian Kasegari 2019 [46]	Randomised controlled trial	Iran	Upper-middle	Direct observation	2019	152	54	35.5	Moderate
Abedadeh-Kalahroudi 2019 [47]	Cross-sectional	Iran	Upper-middle	Direct observation	2015	3239	473	14.6	Low
Ahlberg 2016 [32]	Cross-sectional	Sweden	High	Medical records	2013	596	68	11.4	Moderate
Andrade 2016 [48]	Cross-sectional	Brazil	Upper-middle	Medical records	2014	603	52	9.0	Low
Ashouri 2019 [49]	Cross-sectional	Iran	Upper-middle	Self-reported	2017	600	125	20.8	Low
Banks 2017 [50]	Cross-sectional	Ethiopia	Low	Direct observation	2013	193	22	11.4	Low
Becerra-Chauca 2019 [51]	Cross-sectional	Peru	Upper-middle	Self-reported	2016	250	116	46.4	Low
Biguzzi 2012 [52]	Prospective cohort	Italy	High	Direct observation	2009	6011	1632	27.2	Low
Bohren 2019 [53]	Cross-sectional	Guinea, Myanmar, Ghana and Nigeria	Lower-middle and low	Direct observation	2018	2016	63	3.1	Low
Brandao 2018 [54]	Cross-sectional	Ecuador	Upper middle	Self-reported	2017	252	49	19.4	Low
Burns 2007 [55]	Randomised controlled trial	Italy	High	Direct observation	2003	513	21	4.1	Low
Calik 2018 [19]	Descriptive	Turkey	Upper-middle	Direct observation	2015	351	152	43.3	Low
Chalmers 2009 [56]	Descriptive	Canada	High	Self-reported	2006	5368	805	15.0	Low
Ciriello 2012a [57]	Cross-sectional	Italy	High	Medical records	1996	8112	219	2.7	Low
Ciriello 2012b [57]	Cross-sectional	Italy	High	Medical records	2006	8237	47	0.6	Low
Comas 2017 [58]	Prospective cohort	Spain	High	Direct observation	2013	279	48	17.2	Low
Cortes 2018 [59]	Quasi-experimental before-and-after	Brazil	Upper-middle	Self-reported	2015	140	29	20.7	Low
Cromi 2014 [60]	Cross-sectional	Italy	High	Medical records	Not specified	736	103	14.0	Low
Cuerva 2015 [35]	Prospective cohort	Spain	High	Direct observation	2013	52	36	69.2	Low
da Gama 2016 [61]	Descriptive	Brazil	Upper-middle	Self-reported	2012	11,499	4232	36.8	Low
da Silva Carvalho 2019 [62]	Cross-sectional	Brazil	Upper-middle	Self-reported	2014	314	70	22.3	Low
de Oliveira Peripolli 2019 [63]	Descriptive	Brazil	Upper-middle	Medical records	2015	3078	141	4.5	Low
Dey 2017 [64]	Cross-sectional	India	Lower-middle	Direct observation	2016	875	100	11.4	Low
Dulfe 2016 [65]	Cross-sectional	Brazil	Upper-middle	Self-reported	2014	42	26	61.9	Moderate
Edqvist 2017 [66]	Prospective cohort	Sweden	High	Direct observation	2015	704	16	2.3	Low
Ejegard 2008 [67]	Case–control	Sweden	High	Self-reported	1999	206	39	18.9	Moderate
Fernandes 2017 [68]	Case–control	Brazil	Upper-middle	Medical records	2013	369	12	3.3	Low
Furrer 2015 [14]	Retrospective cohort	Switzerland	High	Medical records	2013	9743	919	9.4	Low
Garcia Cachafeiro 2017 [69]	Cross-sectional	Spain	High	Direct observation	2015	312	49	15.7	Moderate
Hasegawa 2014 [39]	Cross-sectional	Japan	High	Medical records	2012	347,771	38,973	11.2	Moderate
Hasegawa 2020 [40]	Cross-sectional	Japan	High	Medical records	2017	404,444	38,205	9.5	Moderate
Haslinger 2015 [70]	Retrospective cohort	Switzerland	High	Medical records	2011	7832	556	7.1	Low
Hayata 2019 [13]	Cross-sectional	Japan	High	Medical records	2017	1928	265	13.7	Low
Inagaki 2019 [71]	Cross-sectional	Brazil	Upper-middle	Medical records	2016	373	129	34.6	Low
Indraccolo 2016 [72]	Prospective cohort	Italy	High	Direct observation	2015	92	25	27.2	Moderate
Indraccolo 2017 [34]	Prospective cohort	Italy	High	Direct observation	2014	158	41	25.9	Moderate
Iyengar 2009 [42]	Cross-sectional	India	Lower-middle	Self-reported	2006	632	422	67.0	Moderate
Karaçam 2012 [73]	Randomised controlled trial	Turkey	Upper-middle	Direct observation	2009	396	167	42.2	Low
Karacam 2017 [74]	Cross-sectional	Turkey	Upper-middle	Direct observation	2014	303	83	27.4	Low
Kawasoe 2019 [75]	Case–control	Japan	High	Medical records	2016	462	48	10.4	Low
Lazzerini 2018 [76]	Cross-sectional	Italy	High	Self-reported	2018	807	106	13.1	Low
Leal 2019a [77]	Cross-sectional	Brazil	Upper-middle	Not specified	2017	5998	954	15.9	Moderate
Leal 2019b [77]	Cross-sectional	Brazil	Upper-middle	Not specified	2017	1096	235	21.4	Moderate
Lemos 2011 [78]	Cross-sectional	Brazil	Upper-middle	Direct observation	Not specified	33	12	36.4	Moderate
Leombroni 2019 [79]	Cross-sectional	Italy	High	Direct observation	2016	104	31	29.8	Moderate
Lima 2018 [80]	Cross-sectional	Brazil	Upper-middle	Not specified	2014	460	71	15.5	Low
Lopes 2019a [81]	Cross-sectional	Brazil	Upper-middle	Not specified	2012	293	25	8.5	Moderate
Lopes 2019b [81]	Cross-sectional	Brazil	Upper-middle	Not specified	2016	499	61	13.6	Moderate
Martins Franco Motta 2016 [82]	Cross-sectional	Brazil	Upper-middle	Not specified	2013	51	32	62.7	Moderate
Masuda 2020 [5]	Cross-sectional	Philippines	Lower-middle	Direct observation	2018	170	53	31.2	Low
Matsuo 2009 [15]	Cross-sectional	Japan	High	Medical records	2006	661	39	5.9	Low
Maves 2020 [83]	Descriptive	India	Lower-middle	Direct observation	2019	16	11	69.0	Moderate
Mohamed 2017 [84]	Cross-sectional	Egypt	Lower-middle	Direct observation	2017	672	428	63.1	Low
Moiety 2014 [16]	Cross-sectional	Egypt	Lower-middle	Direct observation	2011	8097	1974	24.4	Low
Mollberg 2005 [33]	Cross-sectional	Sweden	High	Medical records	1997	13,716	5236	38.2	Low
Mollberg 2007 [85]	Case–control	Sweden	High	Direct observation	2001	557	90	16.2	Low
Monguilhott 2018 [86]	Cross-sectional	Brazil	Upper-middle	Self-reported	2011	2070	571	27.6	Low
Okumus 2017 [87]	Descriptive	Turkey	Upper-middle	Medical records	2016	240	138	57.5	Moderate
Pazandeh 2015a [88]	Cross-sectional	Iran	Upper-middle	Direct observation	2012	24	16	66.7	Low
Pazandeh 2015b [88]	Cross-sectional	Iran	Upper-middle	Self-reported	2012	100	59	59.0	Low
Pifarotti 2014 [89]	Case–control	Italy	High	Medical records	2010	405	39	9.6	Low
Pinar 2018 [17]	Cross-sectional	Turkey	Upper-middle	Direct observation	2014	350	107	30.6	Low
Prado 2017 [90]	Cross-sectional	Brazil	Upper-middle	Self-reported	2016	456	145	31.7	Low
Raj 2017 [91]	Cross-sectional	India	Lower-middle	Self-reported	2015	2639	211	8.0	Low
Ratcliffe 2016 [92]	Cross-sectional	Tanzania	Low	Direct observation	2014	208	7	3.4	Moderate
Rathfisch 2011 [93]	Descriptive	Turkey	Upper-middle	Direct observation	Not specified	537	245	45.6	Low
Rohde 2016 [94]	Cross-sectional	Portugal	High	Self-reported	2015	468	165	35.0	Moderate
Ruiz de Vinaspre Hernandez 2013 [95]	Retrospective cohort	Spain	High	Medical records	2010	212	71	33.5	Low
Sandin-Bojo 2006 [96]	Cross-sectional	Sweden	High	Medical records	1999	192	25	13.0	Low
Santos 2016 [97]	Quasi-experimental before-and-after	Brazil	Upper-middle	Self-reported	2016	35	2	5.7	Moderate
Sehhati 2013 [98]	Descriptive	Iran	Upper-middle	Not specified	2012	499	153	30.7	Low
Sharma 2019 [99]	Cross-sectional	India	Lower-middle	Direct observation	2015	275	79	29.0	Low
Shimada 2013 [100]	Case–control	Japan	High	Medical records	2012	6317	634	10.0	Low
Skrablin 2011 [101]	Cross-sectional	Croatia	High	Direct observation	2010	205	35	17.1	Low
Sonoda 2012 [102]	Cross-sectional	Japan	High	Medical records	2009	761	68	8.9	Low
Sousa 2016 [103]	Cross-sectional	Brazil	Upper-middle	Not specified	2012	237	22	9.3	Low
Sturzenegger 2017 [104]	Retrospective cohort	Switzerland	High	Medical records	2013	17,957	1447	8.1	Low
Suzuki 2014 [105]	Cross-sectional	Japan	High	Medical records	2012	64	15	24.0	Low
Ukke 2019 [106]	Cross-sectional	Ethiopia	Low	Self-reported	2017	214	35	16.4	Low
Vora 2018 [107]	Cross-sectional	India	Lower-middle	Self-reported	2014	1616	259	16.0	Moderate

**Table 2:** Countries where included studies gathered primary data, income levels per the 2020 World Bank Classification [25]

Country	Number of studies	Number of women included	Income level [25]
*Africa*			
Egypt	2	8769	Lower-middle
Ethiopia	2	407	Low
Tanzania	1	208	Low
*Asia*			
India	6	6053	Lower-middle
Iran	6	4614	Upper-middle
Japan	8	762,408	High
Philippines	1	170	Lower-middle
Turkey	6	2177	Upper-middle
*Europe*			
Croatia	1	205	High
Italy	10	25,175	High
Portugal	1	468	High
Spain	4	855	High
Sweden	6	15,971	High
Switzerland	3	35,532	High
*North America*			
Canada	1	5368	High
*South America*			
Brazil	19	27,596	Upper-middle
Ecuador	1	252	Upper-middle
Peru	1	250	Upper-middle
*Multiple*			
Guinea, Myanmar, Ghana and Nigeria	1	2016	3 lower-middle, 1 low

Of the 80 data sets three were from low income countries, nine from lower-middle income countries, 33 from upper-middle income countries, 34 from high income countries and one included four countries of various income levels

**Table 3:** Summary of characteristics of included studies

Study characteristic	Number of studies	%
*Study design*		
*Observational*		
Case–control	6	7.5
Cross-sectional	51	63.8
Descriptive	8	10.0
Prospective cohort	6	7.5
Retrospective cohort	4	5.0
*Interventional*		
Quasi-experimental before and after	2	2.5
Randomised controlled trial	3	3.8
*Method of measuring fundal pressure*		
Women’s self-report	19	23.6
Direct observation	29	36.3
Medical records	24	30.0
Not specified	8	10.0
*When data were recorded*		
During labour and childbirth care	54	67.5
Postpartum within 6 weeks	17	21.3
Postpartum ranging outside 6 weeks	6	7.5
Not specified	3	3.8

The majority of studies (76/80) comprised women giving birth vaginally or women in labour; two studies included only women undergoing vacuum extraction [32, 33]; one included only women undergoing induction of labour [34]; and one included only women with prolonged second stage of labour [35]. While 29 studies included women with singleton pregnancies only, one study included twin pregnancies only, six included either and 44 did not specify. Most studies either included women of any parity (39/80) or did not specify (29/80), while 11 included nulliparous women only and one included any parity except grandmultiparas (cutoff for this classification not stated). From the 42 studies that reported mean maternal age of the study population, the range was 23 to 30 years. Risk of bias was assessed in all studies (Table 1). Fifty-seven studies were judged to be of high quality, 23 studies were moderate quality and no studies were low quality (Table 4).

**Table 4:** Characteristics of women in the included studies

Characteristics of women	Number of studies	%
*Population*		
Women giving birth vaginally or women in labour	76	95.0
Women undergoing vacuum extraction	2	2.5
Women undergoing induction of labour	1	1.3
Women with prolonged second stage of labour	1	1.3
*Number of fetuses*		
Singleton only	29	36.3
Twins only	1	1.3
Singletons and multiples	6	7.5
Not specified	44	55.0
*Parity*		
Nulliparous women only	11	13.8
Any parity	39	48.8
Any parity except grandmultiparas	1	1.3
Not specified	29	36.3

**Fig. 2:**
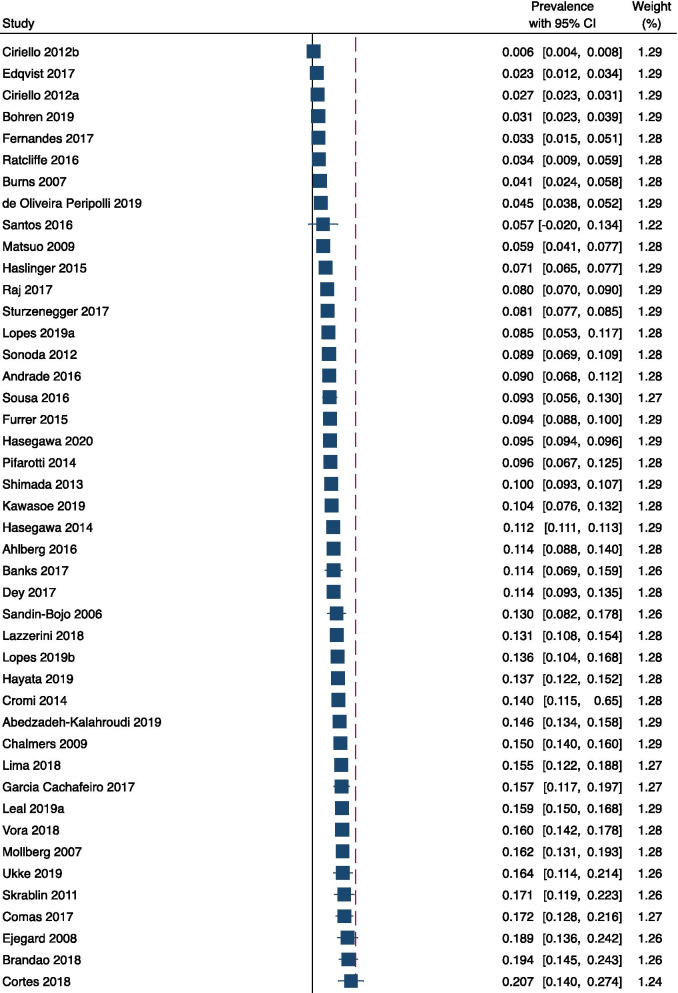

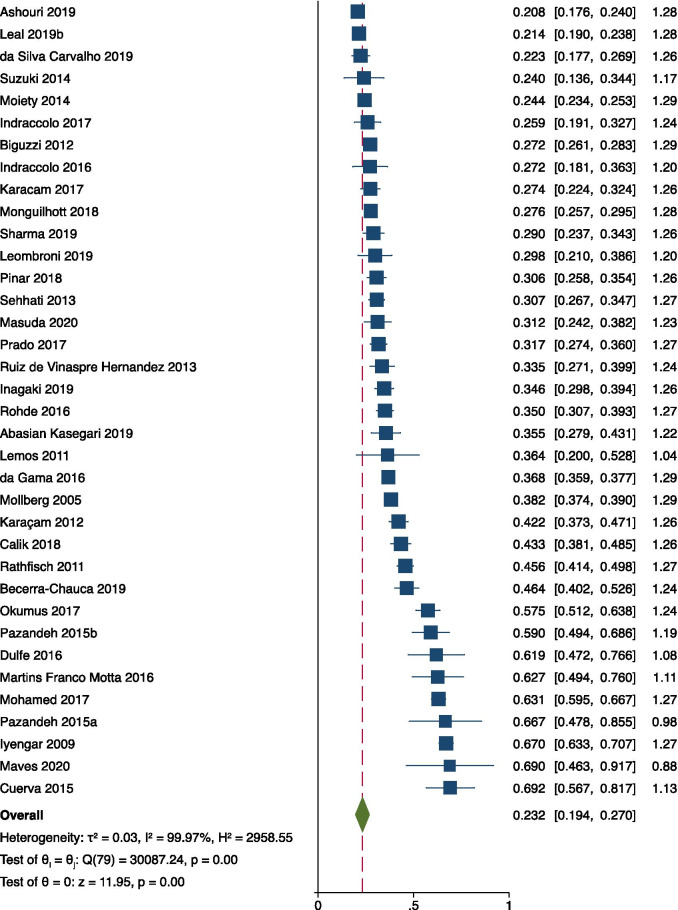
Forest plot of pooled meta-analysis of prevalence of uterine fundal pressure

Eighty data sets from 76 studies (898,544 women) were used for meta-analysis. At a study level, the reported prevalence of fundal pressure ranged from 0.6 to 69.2%. The pooled prevalence was 23.2% (95% CI 19.4–27.0), with high heterogeneity (I^2^ = 99.97%) (Fig. 2). The three sensitivity analyses – excluding studies of low or moderate quality (23 studies excluded), studies with a population less than 500 (44 studies excluded) and studies of populations with limited generalisability (4 studies excluded), respectively – produced results within or close to the confidence interval of the overall meta-analysis, with minimal change in I^2^ (< 0.1%) (Table 5).

**Table 5:** Results of sensitivity analyses

Population	Number of studies	Pooled estimate of prevalence (%, 95% CI)	Heterogeneity (%)
High quality studies only	57	21.5 (17.3–25.7)	99.90
Studies with > 500 women only	36	17.1 (12.0–22.2)	99.98
Study population generalizable to the target population	76	22.6 (18.8–26.4)	99.97

Subgroup analysis of prevalence by country income level was statistically significant (p < 0.001), with highest prevalence in lower-middle income countries (10 studies, 34.7%, 19.0–50.4) and lowest prevalence in low income countries (3 studies, 10.2%, 2.6–17.7) (Table 6). Subgroup analysis of prevalence by method of fundal pressure application was unable to be conducted as it was reported by too few studies. Additional subgroup analyses were performed investigating the prevalence by decade of data collection completion (p = 0.705) and parity (p = 0.098), and did not show significant differences between groups. Subgroup analysis of the method of measuring use of fundal pressure (p = 0.001) showed a statistically significant difference between groups (Table 6). Prevalence values in the women’s self-report and direct observation groups were similar, but a much lower prevalence was recorded in the studies that abstracted data from medical records.

**Table 6:** Results of subgroup analyses

	Number of studies^a^	Pooled estimate of prevalence (%, 95% CI)	*P*
*Income level*	*79*		< 0.001*
High	33	16.4 (12.3–20.5)	
Upper-middle	33	29.0 (23.1–35.0)	
Lower-middle	10	34.7 (19.0–50.4)	
Low	3	10.2 (2.6–17.7)	
*Decade*	*77*		0.705
1991–2000	4	18.2 (3.4–33.0)	
2001–2010	12	20.5 (9.7–31.4)	
2011–2020	61	23.7 (19.3–28.1)	
*Parity*	*22*		0.098
Nulliparous	22	27.3 (18.6–36.0)	
Multiparous	11	15.6 (4.8–26.4)	
*Method of measuring use of fundal pressure*	*72*		0.001*
Direct observation	29	28.0 (21.3–34.8)	
Medical records	24	14.4 (9.1–19.7)	
Women’s self-report	19	29.8 (21.5–38.0)	

^a^One study reporting data from multiple countries of mixed income levels was excluded from the income level subgroup analysis. Studies that did not specify decade of data collection (n = 3) or method of assessing fundal pressure use (n = 8) were not included in subgroup analyses. Twenty-two studies included in subgroup analysis of parity—eleven of these provided data on both nulliparous and multiparous women, and eleven provided data on nulliparous women only

## Discussion

This systematic review and meta-analysis estimated the pooled prevalence of uterine fundal pressure during second stage of labour for women giving birth in health facilities to be 23.2% (19.4–27.0), for studies from 22 countries. Despite significant heterogeneity, the results demonstrate that fundal pressure is widely used. Subgroup analyses suggest this practice may be more common in lower-middle income countries, though there were too few studies to draw conclusions on the use of fundal pressure in low-income countries.

Our findings suggest that studies measuring fundal pressure based on data abstracted from medical records probably under-estimate its use. Previous studies have found that use of fundal pressure is not uniformly recorded or is under-reported in medical records [11, 21]. For example, a study with 18 health care providers in Spain in 2016 found that fundal pressure was often omitted from medical records due to awareness that the procedure was banned and for fear of repercussions [11]. Kline-Kaye and Miller-Stade surveyed 74 institutions in the USA (United States of America) in 1990, and found that only 11% recorded fundal pressure in the clinical file despite 84% of institutions reporting use of the manoeuvre [36]. Reluctance to record the procedure has also been reported by Zaconato et al. and Youssef et al. [37, 38]. This is corroborated by our subgroup analysis, showing that prevalence from medical records were significantly lower than those measured through direct observation and self-reporting. This is an important finding, demonstrating that future studies regarding fundal pressure measurement should not rely on medical records alone.

Despite several exploratory subgroup analyses, we were unable to identify with certainty the source of high heterogeneity in our main results. However, we consider it likely that variation in routine clinical practices and guidelines between different settings was a major contributing factor. While the prevalence of fundal pressure was highest in lower-middle income countries, we identified only three studies (615 women) from low income countries; this limited data may not be representative and further exploration of use of fundal pressure in low income countries is warranted. We have found no published sources providing evidence of use in Australia or the United Kingdom, though a 2006 survey of women in the USA recorded a prevalence of 17% [46]. This survey was excluded from this review due to insufficient information for data extraction, and no sample size was reported. Use of fundal pressure may be linked to geographical region, with intrapartum care practices potentially aligning more with nearby countries rather than being reflective of country income level.

We hypothesised that prevalence of fundal pressure may have declined over the last 20 years, as a result of changes to clinical practice and guidelines reflecting increasing knowledge about benefits and risks over time. Furthermore, courts of law (such is an the USA and European Union) have ruled against the use of the maneuver, which may further discourage its use in some countries due to fear of medico-legal repercussions [7, 21]. These same factors may also lead to under-reporting [11]. Two linked studies conducted in Japan (2012 and 2018) that assessed fundal pressure use based on birth records reported a slight decline in prevalence over time, from 11.2 to 9.5% [39, 40]. This reduction was attributed to lectures and education programs conducted by the Japan Society of Obstetrics and Gynaecology regarding the use of fundal pressure [40]. The subgroup analysis based on decade found no significant difference, and we are unable to conclude whether fundal pressure use has changed over time or not. The rising number of studies published over time may reflect an increasing research interest, and increasing investment in research, rather than increase in fundal pressure use.

The 2017 Cochrane review concluded that there was insufficient evidence of benefit from manual fundal pressure; the preceding version of this review was similarly inconclusive [7, 26]. Both reviews cite the potential for the manoeuvre to cause harm, indicating that evidence regarding safety for the baby is insufficient [7, 26]. Some observational studies have reported increased rates of adverse events following fundal pressure application, such as perineal damage, shoulder dystocia, neonatal fractures and brachial plexus injuries, neonatal hypoxia, lower Apgar scores and higher rates of Neonatal intensive Care Unit admission [13–18]. However, assessing adverse outcomes of fundal pressure using observational methods has limitations, as the indication for fundal pressure may be a pathological scenario that in itself predisposes adverse outcomes. Application of uterine fundal pressure may also impact the woman’s birth experience and perceived quality of care. For example, a 2015 study of 351 women attending a delivery unit in Turkey found that women with fundal pressure had reduced satisfaction with care [19]. Dissatisfaction may be due to discomfort or pain, particularly if the pressure exerted is excessive [7, 11, 17]. Questionnaires of 350 women who received fundal pressure in Turkey in 2014 revealed that 16.5% experienced pain from the procedure [17]. The perceived disruption of the natural birth process may also contribute to women’s dissatisfaction [11]. Reduced maternal satisfaction due to fundal pressure use has been linked with reduced demand for or receptiveness to presence of skilled health personnel in future births, with negative implications for birth outcomes [19, 21].

Not every occurrence of fundal pressure use is harmful; potential harms are possibly relative to the force and duration of pressure applied [12, 21]. Forceful downward pressure on the uterine fundus may be uneven, and can cause rapid changes in intrauterine pressure [12]. If significant downward pressure is exerted toward the maternal spine, vena caval compression and consequent maternal hypotension can occur [21]. Hofmeyr et al. proposed a standardised method of gentle assisted pushing without causing unnecessary discomfort, however this procedure has not been shown to be beneficial [12]. There is likely to be substantial variability in the method of applying fundal pressure, however, data on force and duration of fundal pressure were largely not reported in studies included in this review.

This systematic review employed a broad search strategy across multiple databases to minimise the possibility of missing eligible studies. Two reviewers screened each study and completed data extraction, reducing the chance of errors or introducing individual bias.

There are however a number of limitations. First, although use of fundal pressure in home birth and community settings has been reported [41, 42], we included only studies reporting on women giving birth in health facilities. Therefore, our data cannot be considered as representative at a population level, particularly for countries where a substantial proportion of women give birth in home or community settings. Second, the measurement limitations of individual studies may lead to mis- or under-estimation of fundal pressure prevalence. It is noteworthy that in the two studies that recorded data from both woman’s self-report and direct observation in the same study population there were discrepancies between the reported prevalence. Bohren et al. recorded a prevalence of 3.1% on direct observation and 5.9% on woman’s self-report [53], and Dey et al. recorded 11.4% with direct observation and 1.7% on woman’s self-report [64]. Though, our analysis of studies using more reliable reporting methods (such as direct observation by an independent researcher) was broadly similar to the overall findings. Third, few studies report details of the method of fundal pressure application. We consider it likely that there is variation in positioning, force, and duration of application of fundal pressure between studies. Finally, there may be a publication bias, as settings where fundal pressure is not used are unlikely to publish studies recording and reporting a zero prevalence.

In light of current recommendations internationally, the ongoing use of fundal pressure in health facilities needs to be addressed. In some countries, continued use of fundal pressure may be affected by a lack of resources, where providers use fundal pressure to try and prevent the need for more invasive interventions and associated out of pocket costs [5, 7, 43]. For example, Mishra et al. describe the use of fundal pressure and other bedside interventions to expedite birth in Nepal, as there are insufficient resources to ensure emergency caesarean sections are consistently available in primary and secondary level facilities [43]. Similarly, Masuda et al. stated that fundal pressure is often first line management of women with prolonged second stage in the Philippines, aiming to prevent a need for operative birth and associated costs for the woman [5]. However, interviews with healthcare professionals in the Philippines revealed that those health care providers who used fundal pressure were aware it was not recommended in national guidelines, and continued to use the manoeuvre due to a perceived benefit for women in reducing the duration of second stage based on their clinical experience [5]. Similarly, midwives in Spain demonstrated similar views and awareness of risks [11].

Poor quality, lacking, or inconsistent local and national guidelines may be contributing to the widespread use of fundal pressure. The WHO 2018 recommendation against the use of fundal pressure to facilitate childbirth specified that the Guideline Development Group “had serious concerns about the potential for harm to mother and baby with this procedure” [20]. Local clinicians and policymakers therefore need to ensure that local guidelines align with these evidence-informed international guidelines to optimise maternal care. Protocols and practices at the institutional level should also be reviewed, as there is evidence that these may conflict with national guidelines. For example, whilst the Spanish Ministry of Health advises against fundal pressure, there is evidence demonstrating ongoing use in some Spanish hospitals [11]. Similarly, a survey of policies and procedures at Japanese institutions showed that many did not align with national guidelines [44].

More research is needed to address the ongoing use of uterine fundal pressure. First, local studies assessing prevalence would be beneficial, particularly as part of quality care improvement activities to reduce its use. Second, greater insight into the reasons for fundal pressure use can provide strategies to address this unhelpful but common practice. For example, understanding skilled birth attendants’ awareness of current guidelines, and their reasons for using fundal pressure, would provide insights on how to reduce its use. The application of fundal pressure by family members to assist with a woman’s birth has also been reported [42]. Therefore education may need to be extended into the community, particularly in areas with high rates of home births without skilled health personnel. Additional steps to promote healthcare provider behavioural change include pre-service and in-service training, facilitation and leadership, audit and feedback, barrier identification and quality improvement initiatives [45]. Using these methods, clinicians and policymakers can work toward reducing, and ultimately ending, the use of uterine fundal pressure.

## Conclusion

There is evidence of widespread, ongoing use of manual uterine fundal pressure during labour in health facilities internationally. This procedure currently has no evidence of benefit, and has the potential to cause harm to women and their babies. Efforts to prevent this unnecessary practice should be implemented through development of relevant and evidence-based policies, health professional training, use of audit and feedback, and quality improvement initiatives. Addressing the ongoing use of uterine fundal pressure using these approaches is an important step in optimising intrapartum care for all women.

## Supplementary Information

**Additional file 1.** PRISMA checklist.

**Additional file 2.** Search strategy: the search terms used in MEDLINE, EMBASE, CINAHL and Global Index Medicus databases on 14 February 2020.

**Additional file 3.** Risk of bias tool: an 8-point checklist developed by adapting Rotenstein et al.’s Modified Newcastle–Ottawa Scale and Hoy et al.’s tool for population-based prevalence studies.

**Additional file 4.** Characteristics of included studies: full table presenting the characteristics of included studies, extended version of the table presented on pages 21–24 of the manuscript.

## Data Availability

Extracted data are available in additional files.

## Abbreviations

PRISMA: Preferred Reporting Items for Systematic Reviews and Meta-Analyses
USA: United States of America
WHO: World Health Organization
