# Supplementary material for: The prevalence of uterine fundal pressure during the second stage of labour for women giving birth in health facilities: a systematic review and meta-analysis
Source: Reprod Health. 2021 May 18;18:98. doi: 10.1186/s12978-021-01148-1 (PMC8132352; doi:10.1186/s12978-021-01148-1)
Supplement: Supplementary file 2 — Additional file 2. Search strategy: the search terms used in MEDLINE, EMBASE, CINAHL and Global Index Medicus databases on 14 February 2020. [file 12978_2021_1148_MOESM2_ESM.pdf]

Search Strategy – conducted 14 February 2020

MEDLINE – n=2536

Line	***Search term***	***N***
1	Kristeller*.mp.	46
2	((manual* or direct* or downward or fundal or abdom* or uterine) adj6 (pressur* or push* or force)).mp.	27140
3	((uterine or fundal or abdom*) adj3 massage).mp.	266
4	(abus* and (matern* or wom#n)).mp.	26056
5	1 or 2 or 3 or 4	53471
6	exp Labor Stage, Second/	1378
7	exp Delivery, Obstetric/	78665
8	exp Parturition/	16664
9	(labor or labors or labored or laboring or labour*).mp.	144988
10	(deliver* adj3 vaginal*).mp.	17362
11	(parturition or parturient).mp.	25664
12	(birth or childbirth).mp.	341414
13	intrapartum.mp.	8595
14	6 or 7 or 8 or 9 or 10 or 11 or 12 or 13	510302
15	5 and 14	3745
16	limit 15 to yr="2000 -Current"	**2536**

EMBASE – n=4746

Line	***Search term***	***N***
1	Kristeller*.mp.	74
2	((manual* or direct* or downward or fundal or abdom* or uterine) adj6 (pressur* or push* or force)).mp.	36881
3	((uterine or fundal or abdom*) adj3 massage).mp.	437
4	(abus* and (matern* or wom#n)).mp.	35298
5	1 or 2 or 3 or 4	72637
6	exp labor stage 2/	2202
7	exp obstetric delivery/	142278
8	exp childbirth/	55524
9	(labor or labors or labored or laboring or labour*).mp.	176215
10	(deliver* adj3 vaginal*).mp.	40616
11	(parturition or parturient).mp.	20571
12	(birth or childbirth).mp.	449962
13	intrapartum.mp.	12433

14	6 or 7 or 8 or 9 or 10 or 11 or 12 or 13	654934
15	5 and 14	5967
16	limit 15 to yr="2000 -Current"	**4746**

CINAHL – n=1665

Line	***Search term***	***N***
1	Kristeller*	31
2	((manual* or direct* or downward or fundal or abdom* or uterine) N6 (pressur* or push* or force))	6385
3	((uterine or fundal or abdom*) N3 massage)	165
4	(abus* and (matern* or wom?n))	15998
5	1 or 2 or 3 or 4	22562
6	MH "labor stage, second+"	842
7	MH "delivery, obstetric+"	13215
8	MH "childbirth+"	27197
9	(labor or labors or labored or laboring or labour*)	49775
10	(deliver* N3 vaginal*)	5341
11	(parturition or parturient)	8227
12	(birth or childbirth)	114362
13	intrapartum	4558
14	6 or 7 or 8 or 9 or 10 or 11 or 12 or 13	156983
15	5 and 14	1840
16	limit 15 to yr="2000 -Current"	**1665**

Global Index Medicus – n=225

tw:((tw:((kristeller) OR "manual pressure" OR "fundal pressure" OR "uterine pressure" OR "abdominal pressure" OR "direct pressure" OR "manual pushing" OR "directed pushing" OR "fundal force" OR "abdominal force" OR "fundal massage" OR "uterine massage" OR "abdominal massage" OR (abuse AND (maternal OR maternity OR women)))) AND (tw:(labor OR labors OR labored OR laboring OR labour OR labouring OR laboured OR labours OR parturition OR parturient OR birth OR childbirth OR intrapartum OR "vaginal delivery" OR (mh: ("Labor Stage, Second" OR "Delivery, Obstetric" OR "Parturition"))))) AND (year_cluster:[2000 TO 2020])
