# Supplementary material for: The prevalence of uterine fundal pressure during the second stage of labour for women giving birth in health facilities: a systematic review and meta-analysis
Source: Reprod Health. 2021 May 18;18:98. doi: 10.1186/s12978-021-01148-1 (PMC8132352; doi:10.1186/s12978-021-01148-1)
Supplement: Supplementary file 3 — Additional file 3. Risk of bias tool: an 8-point checklist developed by adapting Rotenstein et al.’s Modified Newcastle–Ottawa Scale and Hoy et al.’s tool for population-based prevalence studies. [file 12978_2021_1148_MOESM3_ESM.pdf]

Risk of Bias Tool

Developed by adapting Rotenstein et al's Modified Newcastle-Ottawa Scale (1) and Hoy et al's tool for population-based prevalence studies (2).

1. **Adequate description of study population (ie. Multiple key parameters described, inclusion/exclusion criteria)**
   Y = study population well described
   N = study population is not well described
2. **Sufficient sample size**
   Y = ≥300 participants
   N = <300 participants
3. **Random selection used**
   Y = a census or some form of random sampling (eg. simple random sampling, stratified random sampling, cluster sampling, systematic sampling)
   N = not a census and random sampling not used
4. **Appropriate temporality of data/observation**
   Y = Observed during labour or puerperium (up to 6 weeks after birth)
   N = Outside labour/puerperium period
5. **Acceptable case definition**
   Y = acceptable case definition used (method, provider)
   N = no acceptable case definition used
6. **Consistent mode of data collection**
   Y = same mode of data collection used for all subjects
   N = same mode of data collection not used for all subjects
7. **Minimisation of likelihood of non-response bias**
   Y = data from direct observation, or in surveys rate of response >75%
   N = rate of response <75%
8. **Were the numerators and denominators appropriate**
   Y = the paper presented appropriate numerator and denominator for the parameter of interest (e.g. the prevalence of fundal pressure)
   N = errors in reporting and/or calculation of numerator and/or denominator, or numerators/denominators not reported

Allocate 1 point for every "Y" – graded out of 8 points:
Low quality (high risk of bias) = 1-2
Moderate quality (moderate risk of bias) = 3-5
High quality (low risk of bias) = 6-8

1. Rotenstein LS, Torre M, Ramos MA, Rosales RC, Guille C, Sen S, et al. Prevalence of Burnout Among Physicians: A Systematic Review. JAMA. 2018;320(11):1131-50.
2. Hoy D, Brooks P, Woolf A, Blyth F, March L, Bain C, et al. Assessing risk of bias in prevalence studies: modification of an existing tool and evidence of interrater agreement. Journal of Clinical Epidemiology. 2012;65(9):934-9.
